# Metabolomics Reveals Differences in Aqueous Humor Composition in Patients With and Without Pseudoexfoliation Syndrome

**DOI:** 10.3389/fmolb.2021.682600

**Published:** 2021-05-14

**Authors:** Diana Anna Dmuchowska, Karolina Pietrowska, Pawel Krasnicki, Tomasz Kowalczyk, Magdalena Misiura, Emil Tomasz Grochowski, Zofia Mariak, Adam Kretowski, Michal Ciborowski

**Affiliations:** ^1^ Department of Ophthalmology, Medical University of Bialystok, Bialystok, Poland; ^2^ Metabolomics Laboratory, Clinical Research Center, Medical University of Bialystok, Bialystok, Poland; ^3^ Department of Pharmaceutical Analysis, Medical University of Bialystok, Bialystok, Poland; ^4^ Department of Endocrinology, Diabetology and Internal Medicine, Medical University of Bialystok, Bialystok, Poland

**Keywords:** ophthalmology, pseudoexfoliation syndrome, aqueous humor, metabolomics, mass spectrometry

## Abstract

Pseudoexfoliation syndrome (XFS) is stress- or inflammation-induced elastosis accompanied by excessive production of microfibrils and their deposition in the anterior segment of the eye. Approximately 60–70 million people are affected by XFS worldwide. It is a component of a systemic disorder, considered a major risk factor for accelerated cataract formation, cataract surgery complications and development of glaucoma, which untreated or inadequately treated may lead to blindness. Moreover, XFS has been associated with cardiovascular and cerebrovascular morbidity, dementia, sensorineural hearing loss and pelvic organ prolapse. The pathogenesis of XFS has not been fully elucidated yet. Aqueous humor (AH) is a transparent fluid filling the anterior and posterior chambers of the eye. Determination of AH metabolites that are characteristic for XFS may provide valuable information about the molecular background of this ocular disorder. The aim of this study was to compare the composition of AH in XFS and non-XFS patients undergoing cataract surgery. The AH samples from 34 patients (15 with XFS and 19 without) were analyzed using liquid chromatography coupled to a Quadrupole Time-of-Flight mass spectrometer (LC-QTOF-MS). The obtained metabolic fingerprints were analyzed using multivariate statistics. Eleven statistically significant metabolites were identified. Compared with the non-XFS group, the AH of patients with XFS contained significantly lower levels of amino acids and their derivatives, for example, arginine (−31%, VIP = 2.38) and homo-arginine (−19%, VIP = 1.38). Also, a decrease in the levels of two acylcarnitines, hydroxybutyrylcarnitine (−29%, VIP = 1.24) and decatrienoylcarnitine (−46%, VIP = 1.89), was observed. However, the level of indoleacetaldehyde in XFS patients was significantly higher (+96%, VIP = 2.64). Other significant metabolites were two well-recognized antioxidants, ascorbic acid (−33%, VIP = 2.11) and hydroxyanthranilic acid (−33%, VIP = 2.25), as well as S-adenosylmethionine, a compound with anti-inflammatory properties (−29%, VIP = 1.93). Metabolic pathway analysis demonstrated that the identified metabolites belonged to eight metabolic pathways, with cysteine and methionine metabolism as well as arginine and proline metabolism being the most frequently represented. XFS can be associated with enhanced oxidative stress and inflammation, as well as with the disturbances of cellular respiration and mitochondrial energy production. Implementation of non-targeted metabolomics provided a better insight into the still not fully understood pathogenesis of XFS.

## Introduction

Pseudoexfoliation is a stress- and inflammation-induced deposition of extracellular fibrillary protein material visible in the anterior segment of the eye. The complex is highly cross-linked and glycosylated. Among others, it contains elastic microfibrillar components, as well as noncollagenous components, such as laminin, nidogen, and fibronectin. The condition is caused by excessive production and reduced degradation of these constituents (Kivelä, 2018; Zenkel, 2018). Aqueous humor (AH) is a transparent fluid filling the anterior and posterior chamber of the eye. AH maintains intraocular pressure and provides nutrients for avascular ocular tissues. It is a mixture of electrolytes, organic solutes, growth factors, cytokines and proteins (Pietrowska et al., 2018a) Pseudoexfoliation syndrome (XFS) can be found on a routine ophthalmic examination. It presents as whitish deposits on the anterior capsule of the crystalline lens and pupil margin. It can also be found throughout the anterior segment of the eye including trabecular meshwork, Schlemm canal, zonules, and ciliary body (Ritch and Schlötzer-Schrehardt, 2001). About 60–70 million people are affected by XFS worldwide. The prevalence of XFS varies across populations, with more frequent occurrence in people of Scandinavian descent. XFS is an age-related progressive condition (Aboobakar et al., 2017). It is considered a major risk factor for accelerated cataract formation, lens subluxation, cataract surgery complications, and the development of glaucoma, which untreated or inadequately treated may lead to blindness (Vazquez-Ferreiro et al., 2016; Vazquez-Ferreiro et al., 2017). Moreover, XFS is a component of a systemic disorder, as similar deposits were found in other (non-ocular) organs, e.g. skin, heart, lungs, liver and kidneys. XFS has been associated with cardiovascular and cerebrovascular morbidity, dementia, sensorineural hearing loss and pelvic organ prolapse (Aboobakar et al., 2017; Aviv et al., 2017; Scharfenberg et al., 2019). It is unclear whether XFS is an ocular condition with systemic implications or systemic disease with ocular manifestation (Aviv et al., 2017). The pathogenesis of XFS is multifactorial and has not been fully elucidated yet. Systemic, environmental and genetic factors have been implicated (Vazquez and Lee, 2014).

Metabolomics provides information about the current biochemical status of a given biological material. Metabolomics analysis of the AH enabled to detect hundreds of molecules and dysregulated metabolic pathways. As metabolomics reflects the phenotype more accurately than many other omics technologies, the results can be more easily translatable to clinical practice (Kell et al., 2005). This is the first study on the metabolomics of AH in XFS. To the best of our knowledge, the metabolomics of AH was the subject of only one published study involving patients with pseudoexfoliation glaucoma (Myer et al., 2020). Moreover, some authors analyzed the proteomics of AH in pseudoexfoliation glaucoma (Kliuchnikova et al., 2016; Sharma et al., 2018; Botling Taube et al., 2019) and plasma metabolomics in XFS (Leruez et al., 2018). The aim of this study was to compare the composition of AH in XFS and non-XFS patients undergoing cataract surgery. Identification of metabolites distinctive for XFS might provide valuable information about the molecular background of this ocular disorder. There is a need for biomarkers to accurately assess the risk of glaucoma, its progression rate and response to treatment in patients with XFS. Identification of such biomarkers would allow for tailored follow-up and treatment of this condition. This is a vitally important objective, given that nearly half of XFS patients will eventually develop pseudoexfoliation glaucoma that if inadequately treated, is a potentially blinding disease (Ritch, 2008).

This study identified novel metabolites related to XFS, as well as metabolic pathways that are disturbed during the course of this condition. These findings contribute to a better understanding of XFS pathophysiology and may help to identify potential novel therapeutic targets.

## Materials and Methods

### Study Participants and Sample Collection

The study included AH samples from 34 patients undergoing cataract surgery. The patients were divided into two groups: with XFS and without (controls). The presence of XFS was assessed on slit-lamp examination. The groups were sex-, age- and BMI-matched. The XFS group included 15 patients (10 women, mean age ±SD = 80.5 ± 5.7 years, mean BMI ±SD = 26.8 ± 3.2 kg/m^2^), and the control group was comprised of 19 patients (11 women, mean age ±SD = 80.1 ± 4.1 years, mean BMI ±SD = 26.4 ± 4.2 kg/m^2^). There were no major differences in systemic comorbidities or medications used (Supplementary Tables S1 and S2). The presence of concomitant ocular disorders and/or diabetes mellitus was an exclusion criterion from the study.

Before the cataract extraction, the anterior chamber of the eye was punctured using a 30 G needle; approximately 50–100 μl of AH was aspirated, transferred to Eppendorf tubes (Eppendorf, Hamburg, Germany), frozen and stored at −80°C until the analysis.

The protocol of the study was reviewed and approved by the Medical Ethics Committee of the Medical University of Bialystok (decisions no. R-I-002/154/2014 and R-I-002/140/2018) and conformed with the provisions of the Declaration of Helsinki. The patients provided their written informed consent to participate in this study.

### Chemicals

Purified water was obtained using the Milli-Q Integral 3 system (Millipore SAS, Molsheim, France). Zomepirac sodium salt (used as an internal standard, IS), L-serine, L-arginine hydrochloride monohydrate, ascorbic acid, L-homoarginine hydrochloride, LC-MS grade methanol acetonitrile, formic acid and LC grade ethanol were purchased from Sigma-Aldrich Chemie GmbH (Steinheim, Germany). Pure p. a. ammonium solution (25%) was purchased from Avantor Performance Materials (Gliwice, Poland). The API-TOF reference mass solution kit (G1969–850001) and tuning solutions, ESI-L low concentration tuning mix (G1969–85000) and ESI-TOF Biopolymer Analysis reference masses (G1969–850003) were purchased from Agilent Technologies (Santa Clara, California, United States).

### Sample Treatment

AH samples were treated as described elsewhere (Pietrowska et al., 2017; Pietrowska et al., 2018b). Briefly, protein precipitation and metabolite extraction were performed by 1-min vortex-mixing of the equal volumes of the AH sample and freeze cold (−20°C) methanol/ethanol (1:1) mixture containing 1 ppm of Zomepirac. Following the extraction, the samples were stored on ice for 10 min and centrifuged at 21,000 × *g* for 20 min at 4°C. The supernatant was filtered through a 0.22 µm nylon filter. Blank extraction followed the same protocol but using the freeze cold (−20°C) methanol/ethanol (1:1) mixture solely.

### Aqueous Humor Metabolic Fingerprinting

The extracted samples were analyzed using an LC-MS system consisting of 1290 Infinity UHPLC (Agilent, Santa Clara, California, United States) with a degasser, two binary pumps and a thermostated autosampler coupled to a 6550 Q-TOF-MS detector (Agilent, Santa Clara, California, United States). The analyses were carried out in a positive (+) and negative (−) ion mode. The samples were analyzed using two different types of chromatography, hydrophilic interaction liquid chromatography (HILIC) for polar compounds and reversed-phase liquid chromatography (RP) for less polar and non-polar compounds. The samples were analyzed against a quality control (QC) sample prepared by mixing several AH samples. The mixture was prepared using some of the samples included in this project and additional samples, as the volume obtained by mixing spare samples from this project was not sufficient. The QC sample was prepared according to the same protocol as the other samples; it was injected ten times at the beginning of the sequence to equilibrate the LC column and later injected again at intervals (every 3–4 samples) to control the stability of the LC-MS system. The samples were analyzed using our standard AH fingerprinting methods (Pietrowska et al., 2017; Pietrowska et al., 2018b). Detailed LC-MS parameters are listed in the Supplementary Material.

### Liquid Chromatography-Mass Spectrometry Data Treatment

The raw data collected by the analytical instrumentation were cleaned of background noise and unrelated ions with the Molecular Feature Extraction (MFE) tool of the Mass Hunter Qualitative Analysis Software B.06.00 (Agilent, Santa Clara, California, United States). The MFE algorithm uses the accuracy of the mass measurements to group ions related by charge-state envelope, isotopic distribution and/or the presence of adducts and dimers. The MFE then creates a list of all possible compounds described by mass, retention time (RT) and abundance. The limit for the background noise for data extraction by the MFE was individually selected for each type of chromatography and each ion mode. The values of 1500, 800, 1200, and 1000 were used for HILIC (+), HILIC (−), RP (+), and RP (−), respectively. The following adduct settings: +H, +Na, +K for positive ion mode and −H, +HCOO, +Cl for negative ion mode were applied to identify the co-eluting adducts of the same feature. Dehydration neutral losses were also allowed. Additionally, +NH4 was included in the list of possible adducts for data recorded in HILIC ESI + mode. Only metabolic features with a quality score ≥80% were accepted to preserve a good quality of the data. The sample alignment, as well as data cleaning and filtering, were performed using Mass Profiler Professional 12.6.1 (Agilent, Santa Clara, California, United States). The parameters applied for the alignment were 1% for RT and 15 ppm for the mass variation. During the first step of the data treatment, the signals present in the blank sample were separated from the signals present in the biological samples by the use of the Venn diagram. Before the statistical analysis, a quality assurance (QA) protocol was implemented to keep solely the repetitively measured metabolic features. As the QC sample was prepared by mixing additional AH samples, not only metabolic features with CV <30% but also those absent in QC samples were accepted. Additionally, the features were filtered to keep only those present in at least 80% of the samples in at least one of the studied groups. Missing values were replaced as described by Armitage et al. (2015).

### Statistical Analysis

Principal component analysis (PCA) was used to check the quality of the data (clustering of the QC samples) and to detect potential outliers. A multivariate statistical analysis based on the orthogonal partial least squares discriminant analysis (OPLS-DA) models was carried out to identify the metabolites that discriminated the XFS group from the controls. The validity of the models was evaluated based on the results of a permutation test and *p*-value provided by cross-validated analysis of variance (CV-ANOVA). Additionally, to assess the predictive accuracy of the OPLS-DA models, for each model receiver operating characteristic (ROC) analysis was performed. The contribution of each metabolite to the observed sample discrimination was assessed based on the volcano plots obtained by plotting variable importance in the projection (VIP) against loading values scaled as correlation coefficient values [*p*(corr)] generated based on the obtained OPLS-DA models. Variables with VIP >1.0 and absolute *p*(corr) >0.4 were considered significant. Multivariate calculations and plots were obtained with SIMCA−P + 13.0.3.0 (Umetrics, Umeå, Sweden) or with SIMCA 17 (Sartorius Stedim Data Analytics AB, Gottingen, Germany). Additionally, for each metabolite *p*-value was calculated using t-test or Mann-Whitney nonparametric U test, depending on the normality of the data distribution (assessed by the Shapiro-Wilk test). Obtained *p*-values were corrected by Benjamini-Hochberg false discovery rate (FDR).

### Metabolite Identification

The metabolites were identified based on the MS/MS fragmentation, as described previously (Pietrowska et al., 2017). Accurate masses of features were searched against the METLIN, KEGG, LIPIDMAPS, and HMDB databases, accessed simultaneously by CEU Mass Mediator (an on-line search engine, http://ceumass.eps.uspceu.es/mediator/). Putative identities were then confirmed by matching the experimental MS/MS spectra with the MS/MS spectra from the databases or with the fragmentation spectra and retention times obtained for the metabolite’s standard. The experiments were repeated under identical chromatographic conditions as the primary analysis. Ions were targeted for collision-induced dissociation (CID) fragmentation on the fly based on the previously determined accurate mass and retention time. The identity of carnitines was confirmed based on the already described fragmentation pattern (Piszcz et al., 2016).

### Metabolic Pathway Analysis

The pathway analysis was performed with MetaboAnalyst 4.0 (http://www.metaboanalyst.ca/). This on-line tool analyses the impact of particular compounds on biochemical pathways specifically for metabolomics studies (Chong et al., 2018).

## Results

**FIGURE 1:**
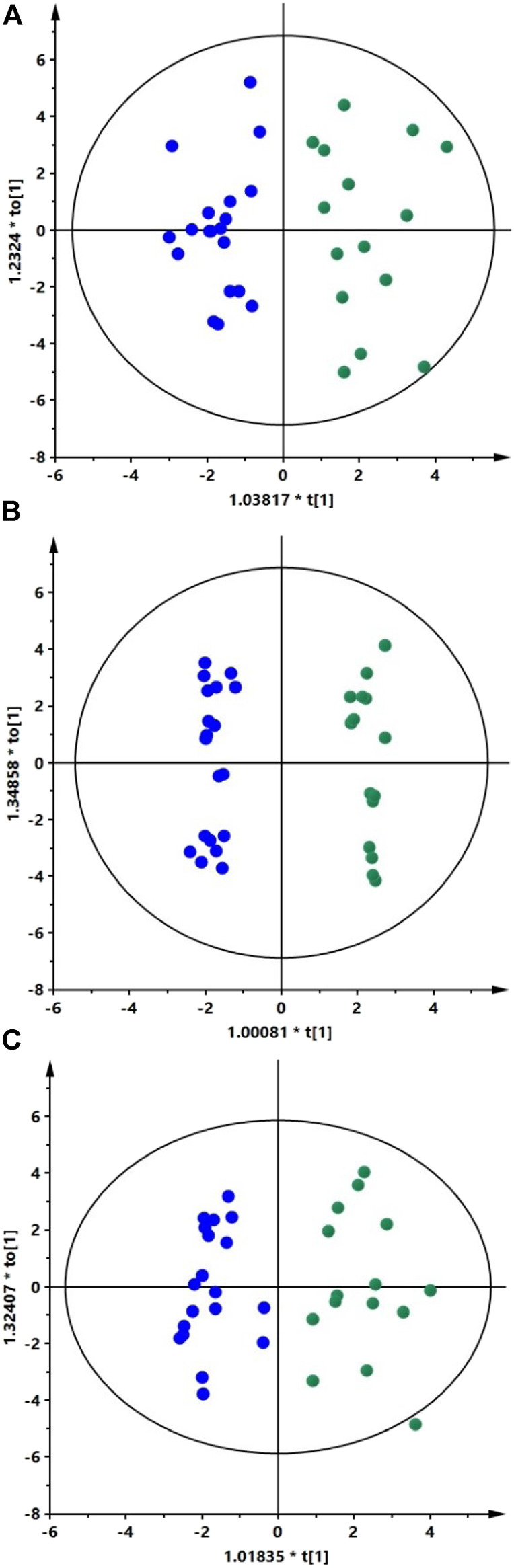
Classification of XFS patients and controls based on AH metabolic fingerprinting data.

We analyzed AH samples from cataract patients with XFS (*n* = 15) and without (*n* = 19). The metabolites in extracted AH samples were separated by two types of liquid chromatography (RP and HILIC) and then detected with a QTOF mass analyzer. Four datasets were obtained with 296, 120, 211, and 150 metabolic features for HILIC (+), HILIC (−), RP (+) and RP (−), respectively. PCA models were obtained to verify the quality of the obtained data. Clustering of the QC samples (Supplementary Figure S1) indicated the proper quality of the data. OPLS-DA models were used to identify statistically significant metabolites differentiating XFS patients from the controls. The models were obtained for each dataset (Figure 1). The study groups could not be differentiated based on the negative ion mode data obtained using RP chromatography.

The OPLS-DA scatter plots show a clear distinction between patients with XFS (green dots) and the controls (blue dots). Panels A, B, and C show models obtained for the HILIC (+) data (R2 = 0.635, Q2 = 0.345), HILIC (−) data (R2 = 0.559, Q2 = 0.471) and RP (+) data (R2 = 0.624, Q2 = 0.417), respectively. The results of permutation tests and CV-ANOVA showed that models were statistically valid. For each model obtained Q2 intercept values and *p*-values were as follow: HILIC (+) data (Q2 = −0.342, *p* = 0.01), HILIC (−) data (Q2 = −0.616, *p* = 0.0008), and RP (+) data (Q2 = −0.345, *p* = 0.008). AUC values obtained for all three models based on ROC analysis were equal to 1.0, indicating perfect predictive accuracy.

**FIGURE 2:**
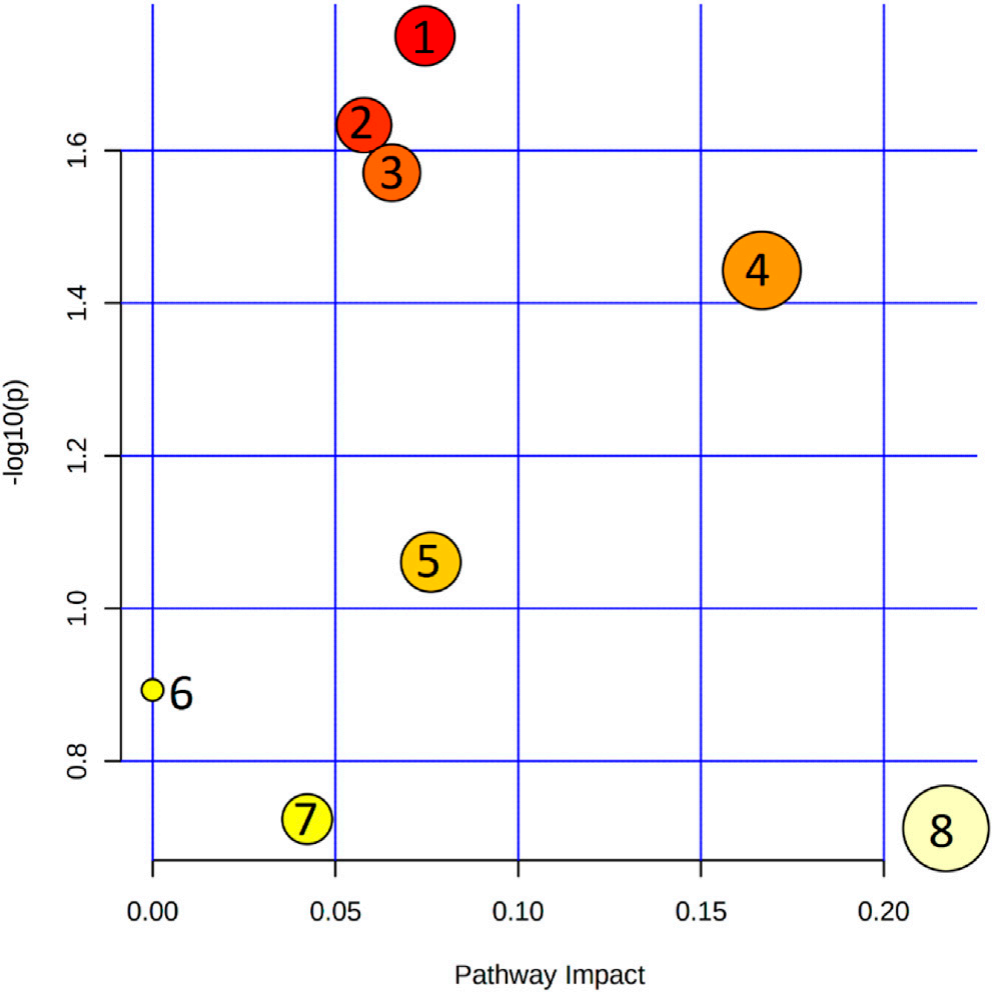
Pathway analysis of metabolites that were shown to differ significantly between the metabolic profiles of aqueous humor in patients with XFS and without. 1. Cysteine and methionine metabolism; 2. Arginine and proline metabolism; 3. Tryptophan metabolism; 4. Aminoacyl-tRNA biosynthesis; 5. Arginine biosynthesis; 6. Sphingolipid metabolism; 7. Glyoxylate and dicarboxylate metabolism; 8. Glycine, serine and threonine metabolism.

Eleven metabolites (Table 1; Supplementary Table S3) were shown to discriminate significantly patients with XFS and the controls. Compared with the controls, AH of patients with XFS contained significantly lower levels of amino acids, organic acids and acylcarnitines. Indoleacetaldehyde was the only metabolite, the level of which was significantly higher in XFS patients than in the controls. Biochemical pathway analysis involving the significant metabolites mentioned above identified several altered pathways in the AH of XFS patients, especially the metabolism of amino acids and aminoacyl-tRNA biosynthesis (Figure 2). Among other alterations, the disturbances of tryptophan metabolism were found in XFS patients, with a resultant decrease in 3-hydroxyanthranilic acid level and an increase in indoleacetaldehyde level.

**TABLE 1:** Metabolites differentiating significantly AH of patients with XFS from AH of non-XFS controls.

Name	Change (%)	*p*(corr)	VIP	*p*-value	Corrected *p*-value	Monoisotopic mass [Da]
L-serine*	−30.76	−0.44	1.01	0.02	0.07	105.0426
3-hydroxy anthranilic acid	−33.10	−0.50	2.25	0.05	0.3	153.0426
	−38.22	−0.40	2.12	0.002	0.2	153.0426
Indoleacetaldehyde	+96.36	0.64	2.64	0.006	0.2	159.0684
2-hydroxycinnamic acid/m-coumaric acid (co-elution)	−25.24	−0.51	1.23	0.01	0.2	164.0473
L-arginine*	−30.68	−0.59	2.38	0.04	0.3	174.1117
Ascorbic acid*	−32.81	−0.50	2.11	0.02	0.3	176.0321
Homo-L-arginine*	−18.72	−0.41	1.38	0.01	0.2	188.1273
Ergothioneine	−66.42	−0.41	1.81	0.05	0.3	229.0884
	−64.76	−0.44	2.48	0.03	0.3	229.0885
Hydroxybutyrylcarnitine	−28.86	−0.46	1.24	0.02	0.2	247.142
Decatrienoylcarnitine	−45.83	−0.45	1.89	0.002	0.09	309.194
S-adenosyl-L-methioninate	−29.15	−0.59	1.93	0.0004	0.04	398.1372

The direction of change indicates increased (+) or decreased (−) abundance of a metabolite in the AH of patients with XFS in comparison to its abundance in the AH of the controls. The VIP values were calculated based on OPLS-DA models built separately for each method and ion mode.

* The identity of these metabolites was confirmed by the LC-MS/MS analysis of the standard.

## Discussion

The aim of this study was to identify potential differences in the metabolic composition of AH obtained from patients with and without XFS. We focused on XFS, rather than on its consequence, pseudoexfoliation glaucoma, to characterize an initial pathological stage of the latter condition. Our findings imply that XFS is associated with enhanced oxidative stress and inflammation, as well as with the disruption of cellular respiration and mitochondrial energy production. Gut microbiota might also play a role in the pathogenesis of this condition. These observations are consistent with the results of previous studies (Koliakos et al., 2008; Erdurmuş et al., 2011; Zenkel et al., 2011; Aboobakar et al., 2017; Borrás, 2018; Papadopoulou et al., 2018; Schlötzer-Schrehardt, 2018; Sharma et al., 2018; Botling Taube et al., 2019; Ghaffari Sharaf et al., 2020).

To the best of our knowledge, only one metabolomics study of AH from patients with pseudoexfoliation glaucoma, not XFS, has been conducted thus far (Myer et al., 2020). Similarly to Myer et al. (2020), we have found decreased levels of L-arginine. Interestingly, according to Leruez et al. neither arginine nor tryptophan proved to be significant in the plasma of XFS (Leruez et al., 2018), which implies that the metabolism of these amino acids in XFS is affected locally, in the anterior chamber of the eye, rather than systemically. L-arginine is utilized by NO synthases for the synthesis of NO (Stuehr, 2004). Another substrate for NO synthase is Homo-L-arginine, which in this study was also found decreased in AH of patients with XFS (Tsikas and Wu, 2015). Thus, the reduction of arginine and homoarginine levels in patients with XFS points to a dysfunction of the NO pathway, which plays a significant role in regulating AH outflow balance in the eye (Kotikoski et al., 2002; Chang et al., 2015) and seems to be involved in the pathogenesis of glaucoma (Polak et al., 2007). Lower levels of NO in AH of XFS patients with glaucoma have already been reported by Kotikoski et al. (2002). L-arginine has also antioxidant and anti-inflammatory properties (Jobgen et al., 2006; Thomas et al., 2008; Tosun et al., 2012; Pimentel et al., 2013; Wang et al., 2014; Erdinest et al., 2015; Park et al., 2020).

As an important one-carbon donor to the folate cycle, serine contributes to nucleotide synthesis, methylation reactions and the antioxidant defense (Bleich et al., 2004). Folate deficiency is a risk factor for XFS (Aboobakar et al., 2017). A decreased level of serine might be associated with a reduced concentration of S-adenosyl-methionine. S-adenosyl-methionine has anti-inflammatory activity and is an intermediate in the homocysteine biosynthesis pathway. Its decreased level in patients with XFS might reflect the increased activity of the methionine cycle and excessive production of homocysteine. Although homocysteine was not identified as a significant metabolite in the present study, it has been well-documented in both plasma and AH of patients with XFS. It is unclear whether hyperhomocysteinemia is a cause or consequence of XFS (Bleich et al., 2004; Aboobakar et al., 2017; Leruez et al., 2018). According to Rebecca et al. hyperhomocysteinemia in XFS causes structural changes promoting elastin aggregation (Rebecca et al., 2019). Serine metabolism also intersects with the methionine cycle (Yang and Vousden, 2016), in which methionine synthase remethylates homocysteine in a vitamin B12-dependent reaction (Yang and Vousden, 2016; Koc and Kaya, 2020). It is suggested that the intake of vitamin B6 and vitamin B12 may reduce the risk of pseudoexfoliation glaucoma (Kang et al., 2014). This observation might support the theory about the increased activity of the methionine pathway and a deficit of serine and S-adenosyl-methionine in patients with XFS.

In line with Koliakos et al. (2003) we have found decreased level of ascorbic acid, a potent antioxidant. It implies that XFS is associated with enhanced oxidative stress within the eye. Furthermore, vitamin C supplementation is believed to protect against the XFS progression (Koliakos et al., 2003). Due to the reduced concentration of ascorbic acid in AH, patients with XFS might be protected less against UV radiation. This hypothesis is supported by the observation that prolonged time spent outdoors is an independent risk factor for the development of XFS (Kang et al., 2012; Pasquale et al., 2014). The role of vitamin C in cataract prevention was described by Weikel et al. (2014). Aside from the reduced levels of ascorbic acid, we also found decreased levels of 3-hydroxyanthranilic acid in the AH of XFS patients. The latter is one of the tryptophan-derived compounds that act as physical filters for bands from the UVA spectrum (Wilson et al., 2016). UV radiation is implicated in the photodamage to the human eye. Decreased levels of tryptophan-derived constituents impair protection from UV light, lead to enhanced oxidative damage and accumulation of modified proteins implicated in nuclear cataract formation (Tweeddale et al., 2016). Moreover, Laganovska et al. suggested that XFS might be associated with disturbances in the kynurenine pathway, the primary route for tryptophan catabolism (Laganovska et al., 2003). This might lead to the enhancement of oxidative stress in the eye, a key factor in the pathogenesis of ocular diseases (Kruk et al., 2015). Chronic oxidative stress may disrupt the balance between matrix metalloproteinases and their tissue inhibitors, which leads to the accumulation of extracellular matrix fibrils constituting pseudoexfoliation material (Zenkel et al., 2011; Schlötzer-Schrehardt, 2012). Enhanced oxidative stress in the eye was also reported by other authors, along with mitochondrial dysfunction (Koliakos et al., 2008; Erdurmuş et al., 2011; Zenkel et al., 2011; Borrás, 2018; Papadopoulou et al., 2018; Schlötzer-Schrehardt, 2018; Sharma et al., 2018; Botling Taube et al., 2019; Ghaffari Sharaf et al., 2020). The latter might play a role in XFS progression. Non-functioning mitochondria were observed *in vitro* models of XFS; the mitochondrial dysfunction might negatively affect the efficiency of respiration, leading to a decrease in ATP levels and enhanced synthesis of reactive oxygen species (Want et al., 2016). Acylcarnitines are involved in the mitochondrial metabolism of lipids and fatty acids and are essential for the proper function of the eye (Pescosolido et al., 2008; Pescosolido et al., 2009). Carnitines may protect against selenite-induced cataract, and their loss may be a marker of disease development (Ritch, 2007). As postulated recently, the reduced level of carnitines in XFS might reflect mitochondrial dysfunction (Want et al., 2016; Ghaffari Sharaf et al., 2020). Our findings seem to support this theory, as the levels of hydroxybutyrylcarnitine and decatrienoylcarnitine in XFS patients were significantly lower than in the controls. The deficit of carnitines in the AH might reflect enhanced β-oxidation and elevated levels of mitochondrial acetyl-CoA, conditions that eventually lead to the development of cellular oxidative stress (Schönfeld et al., 2010). In summary, we have demonstrated that the AH of XFS patients shows a decreased antioxidant content and increased oxidative stress factors.

Some of the metabolites identified in this study (e.g. indoleacetaldehyde, 2-hydroxycinnamic acid/m-coumaric acid, ergothioneine) might be related to the human microbiome, especially the intestinal microflora (Filannino et al., 2014; Cumming et al., 2018; Gao et al., 2018). Several authors suggested a link between gut microbiota and eye diseases (Lin, 2018; Shivaji, 2019; Petrillo et al., 2020). AH from patients with XFS contained significantly higher levels of indoleacetaldehyde, a precursor in the bacterial synthesis of indoleacetic acid from tryptophan (Gao et al., 2018; Roager and Licht, 2018). An elevated level of indoleacetic acid in mammalian cells may cause many disorders, such as disruption of apoptosis, protein degradation and cell cycle progression arrest (Holland et al., 2012; Zhao et al., 2015). While the function of ergothioneine in mammalian cells is still not fully understood, recent evidence suggests that this metabolite is a powerful antioxidant; cells deficient in ergothioneine were shown to be more prone to oxidative stress, with resultant increased damage to mitochondrial DNA, protein oxidation and peroxidation of lipids (Paul and Snyder, 2010; Cheah and Halliwell, 2012). Hence, the deficit of ergothioneine in the AH of patients with XFS seems to support the notion that this condition is associated with enhanced oxidative stress in the eye.

This study had several potential limitations. The sample size was relatively small and from a limited geographical area. Hence, it is unclear whether the results could be generalized to other ethnic groups, especially considering the uneven distribution of XFS globally. Moreover, the study focused solely on XFS rather than on pseudoexfoliation glaucoma. Finally, not all metabolites could be analyzed with the LC-MS approach.

An interesting direction of future research would be a comparative analysis of the AH and plasma metabolomics in the same patients, especially given that XFS is a systemic condition associated with increased vascular permeability of the blood-aqueous barrier (Scharfenberg et al., 2019). From a clinical perspective, the results of this study might justify further research on the anti-oxidative and anti-inflammatory treatment of XFS.

## Conclusion

The results of this study suggest that the pathogenesis of XFS may involve enhanced oxidative stress and inflammation, as well as the dysfunction of mitochondria and gut microbiota. The knowledge of metabolites and metabolic pathways involved in XFS pathogenesis might facilitate the development of novel prevention and treatment strategies.

## Data Availability

The raw data supporting the conclusion of this article will be made available by the authors, without undue reservation.
