# Supplementary material for: Metabolomics Reveals Differences in Aqueous Humor Composition in Patients With and Without Pseudoexfoliation Syndrome
Source: Front Mol Biosci. 2021 May 14;8:682600. doi: 10.3389/fmolb.2021.682600 (PMC8160430; doi:10.3389/fmolb.2021.682600)
Supplement: Supplementary file 1 [file DataSheet1.docx]

Supporting information to: “**Metabolomics reveals differences in aqueous humor composition in patients with and without pseudoexfoliation syndrome”**

Diana Anna Dmuchowska^1^*, Karolina Pietrowska^2^, Pawel Krasnicki^1^, Tomasz Kowalczyk^2^, Magdalena Misiura^3^, Emil Tomasz Grochowski¹, Zofia Mariak¹, Adam Kretowski^2,4^, Michal Ciborowski^2^*

^1^Department of Ophthalmology, Medical University of Bialystok, Bialystok, Poland

^2^Metabolomics Laboratory, Clinical Research Centre, Medical University of Bialystok, Bialystok, Poland

^3^Department of Pharmaceutical Analysis, Medical University of Bialystok, Bialystok, Poland

^4^Department of Endocrinology, Diabetology and Internal Medicine, Medical University of Bialystok, Bialystok, Poland

🖂 Dr. Michal Ciborowski, Metabolomics Laboratory, Clinical Research Centre, Medical University of Bialystok, Poland, michal.ciborowski@umb.edu.pl

🖂 Dr. Diana Anna Dmuchowska, Department of Ophthalmology, Medical University of Bialystok, Bialystok, Poland, diana.dmuchowska@umb.edu.pl

1. LC-RP-MS analysis

2 𝜇L of extracted AH sample was injected into a thermostated (30°C) RP Poroshell 120 EC-C18,3.0 × 100 mm, 2.7 𝜇m column (Agilent Technologies,Santa Clara, California, USA). The flow rate was 0.5 mL/min with solvent A (water with 0.1% formic acid) and solvent B (acetonitrile with 0.1% formic acid). The gradient started at 1% phase B for the first minute and was increasing to reach 100% of phase B in 10 min. After that, the gradient returned to initial conditions (1% of phase B) in 0.1 min and was maintained at this solvents proportion for 4.9 min in order to re-equilibrate the system for the next injection.

The mass spectrometer was operated in full scan mode from m/z 50 – 1000. The capillary voltage was set to 3kV; the drying gas flow rate was 12L/min at 250°C and gas nebulizer at 45 psig; fragmentor voltage was 225V for positive and 275V for negative ionization mode. Data was collected in centroid mode at a scan rate of 2 spectra per second. Accurate mass measurements were obtained by means of calibrant solution delivery using a dual-nebulizer ESI source. A calibrating solution (G1969-85000) containing reference masses at m/z 121.0509 (protonated purine) and m/z 922.0098 (protonated hexakis (1H,1H,3H-tetrafluoropropoxy)phosphazine or HP-921) in positive ion mode or m/z 119.0363 (proton abstracted purine) and m/z 966.0007 (formate adduct of HP-921) in negative ionmode was continuously introduced by an isocratic pump (Agilent, Santa Clara, California, USA) at a flow rate of 0.5 mL/min (1 : 100 split).

1. LC-HILIC-MS analysis

Extracted sample (1μL) was injected into a thermostated (30°C) SeQuant® PEEK ZIC®- HILIC 2.1x100 mm, 3.5µm column (Merk KGaA, Darmstadt, Germany). The flow rate was 0.1 mL/min with solvent A (5mM ammonium formate in water, pH=4) and solvent B (acetonitrile with 0.1% formic acid). The gradient started at 80% phase B and was decreasing to reach 15% of phase B in 13 min. After that, the gradient returned to starting conditions (80% of phase B) in 1 min and was maintained at this solvents proportion for 5 min in order to re-equilibrate the system for the next injection.

The mass spectrometer was operated in full scan mode from m/z 50–1000. The capillary voltage was set to 3.5 kV; the drying gas flow rate was 13L/min at 200°C and gas nebulizer at 30 psig; fragmentor voltage was 225V for both ionization modes. Data were collected in centroid mode at a scan rate of 1.5 spectra per second. Accurate mass measurements were obtained by means of calibrant solution delivery using a dual-nebulizer ESI source. A calibrating solution (G1969-85000) containing reference masses at m/z 121.0509 (protonated purine) and m/z 922.0098 (protonated hexakis (1H,1H,3H-tetrafluoropropoxy)phosphazine or HP-921) in positive ion mode or m/z 112.9856 (TFA anion) and m/z 966.0007 (formate adduct of HP-921) in negative ion mode was continuously introduced by an isocratic pump (Agilent, Santa Clara, California, USA) at a flow rate of 0.4 mL/min (1:100 split).


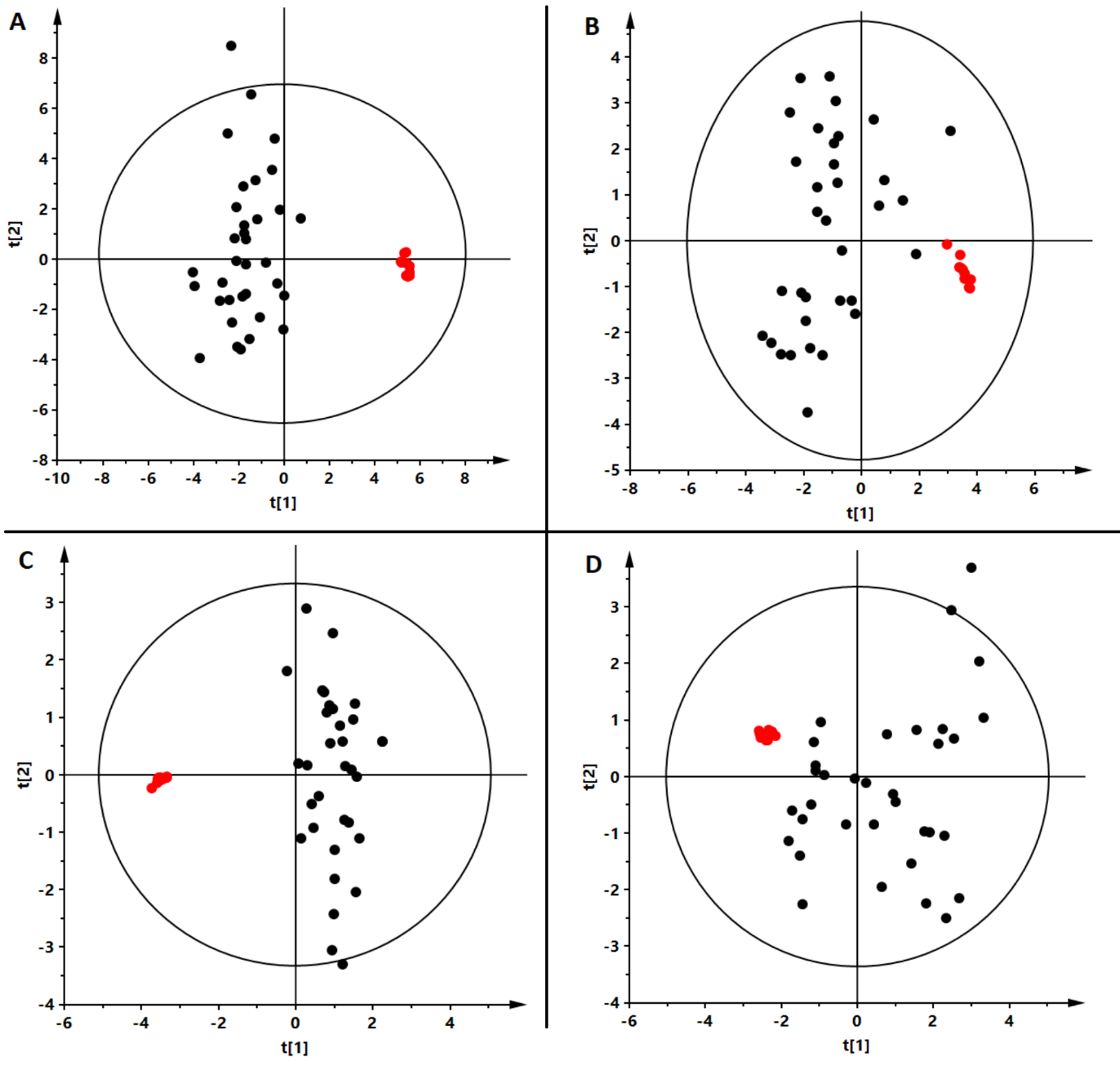


Figure S1. PCA plots performed for recorded datasets.

The PCA plots show the classification of QC samples (red dots) in both types of chromatography (RP and HILIC) and both ion modes (positive and negative). Black dots represent studied AH samples. Panel A shows RP (+) data, panel B - RP (-), panel C - HILIC (+), and panel D - HILIC (-).

Table S1. Baseline characteristics of patients with pseudoexfoliation syndrome.

| Number | Age (years) | Gender | BMI | Type of cataract | Comorbidities | Other medications |
| --- | --- | --- | --- | --- | --- | --- |
| 1 | 86 | Female | 22.9 | Nuclear/Subcapsular | none | none |
| 2 | 85 | Male | 25.0 | Nuclear/Cortical | Hypertension, Benign Prostatic Hyperplasia | Bisoprololi fumaras |
| 3 | 66 | Female | 33.5 | Nuclear/Cortical/Subcapsular | Hypertension, Hyperlipidemia | Acidum acetylsalicylicum, Indapamidum, Bisoprololi fumaras, Atorvastatinum |
| 4 | 79 | Male | 29.8 | Nuclear | Hypertension, Coronary Artery Disease, Chronic Obstructive Pulmonary Disease, history of stroke | Acenocumarolum, Propafenoni hydrochloridum, Torasemidum, Metoprololi tartras, Formoteroli fumaras dihydricus, Tiotropium, Telmisartanum |
| 5 | 83 | Female | 22.5 | Nuclear | Hypertension, Coronary Artery Disease, Chronic Obstructive Pulmonary Disease, Atrial Fibrillation | Carvedilolum, Bisoprololi fumaras, Acidum acetylsalicylicum, Pentoxifyllinum, Doxazosinum, Theophyllinum, Ipratropii bromidum |
| 6 | 82 | Female | 30.1 | Nuclear/Cortical | Hypertension, Coronary Artery Disease, Chronic Obstructive Pulmonary Disease, Hyperlipidemia | Perindoprilum argininum, Acidum acetylsalicylicum, Nebivololum, Pantoprazolum, Furosemidum, Simvastatinum, Salmeterolum. |
| 7 | 86 | Male | 22.5 | Nuclear/Cortical | Hypertension, Coronary Artery Disease, Benign Prostatic Hyperplasia | unknown |
| 8 | 83 | Female | 25.4 | Nuclear | Hypertension | Atorvastatinum, Valsartanum, Hydrochlorothiazidum |
| 9 | 72 | Female | 27.9 | Nuclear/Cortical/Subcapsular | Hypertension | unknown |
| 10 | 83 | Female | 27.1 | Nuclear | none | none |
| 11 | 79 | Male | 27.0 | Nuclear/Cortical | Hypertension, Coronary Artery Disease, Hyperlipidemia | Ramiprilum, Atorvastatinum, Indapamidum, Amlodipinum, Ciclesonidum, Montelukastum, Doxazosinum, Bisoprololi fumaras, Clopidogrelum, Pantoprazolum. |
| 12 | 87 | Female | 31.3 | Nuclear/Cortical/Subcapsular | Hypertension, Asthma, history of Coronary Artery Bypass Graft | Amlodipinum |
| 13 | 82 | Female | 27.3 | Nuclear/Cortical | Hypertension | unknown |
| 14 | 81 | Male | 27.7 | Nuclear | Coronary Artery Disease, Hyperlipidemia | Carvedilolum, Rosuvastatinum, Ramiprilum, Acidum acetylsalicylicum. |
| 15 | 84 | Female | 22.6 | Nuclear/Cortical | Hypertension, Coronary Artery Disease, Chronic Obstructive Pulmonary Disease, Goiter, history of Pacemaker Implantation | Valsartanum, Hydrochlorothiazidum, Carvedilolum, Piracetamum, Cholecalciferolum, Meloxicamum |

Standard topical treatment before cataract surgery included: Ofloxacinum/Moxifloxacinum, Proxymetacaini hydrochloridum, Tropicamidum, Phenylephrinum, Timololum, Povidonum iodinatum and Hydroxyzini hydrochloridum orally.

Table S2. Baseline characteristics of the control group.

| Number | Age (years) | Gender | BMI | Type of cataract | Comorbidities | Other medications |
| --- | --- | --- | --- | --- | --- | --- |
| 1 | 86 | Female | 25.4 | Nuclear/Subcapsular | Coronary Artery Disease, Deep Vein Thrombosis | Bisoprololi fumaras |
| 2 | 85 | Male | 22.8 | Nuclear | Hypertension, Benign Prostatic Hyperplasia | Indapamidum, Finasteridum, Pantoprazolum. |
| 3 | 84 | Male | 29.3 | Nuclear/Subcapsular | Chronic Obstructive Pulmonary Disease, Benign Prostatic Hyperplasia | Pentoxifyllinum, Doxazosinum, Theophyllinum, Ipratropii bromidum,Tolterodini hydrogenotartras, Vinpocetinum |
| 4 | 84 | Female | 24.2 | Nuclear/Cortical | Hypertension | Amlodipinum, Betahistini dihydrochloridum |
| 5 | 84 | Female | 26.1 | Nuclear/Cortical | Hypertension, Coronary Artery Disease, Hyperlipidemia | Spironolactonum, Bisoprololi fumaras, Acidum acetylsalicylicum, Atorvastatinum, Vinpocetinum |
| 6 | 84 | Male | 30.6 | Nuclear/Cortical/Subcapsular | Hypertension, Chronic Obstructive Pulmonary Disease, Asthma, Hyperlipidemia | Pentoxifyllinum, Ramiprilum, Amlodipinum, Acidum acetylsalicylicum, Tamsulosini hydrochloridum, Tolterodini tartras, Atorvastatinum |
| 7 | 83 | Female | 22.2 | Nuclear/Cortical | Coronary Artery Disease | Bisoprololi fumaras |
| 8 | 83 | Male | 29.4 | Nuclear | Hypertension, Coronary Artery Disease, Benign Prostatic Hyperplasia | Finasteridum, Metoprololi tartras, Isosorbidi mononitras, Terazosinum |
| 9 | 81 | Female | 28.8 | Nuclear/Cortical | Hypertension, Chronic Obstructive Pulmonary Disease, Hyperlipidemia, Chronic Venous Insufficiency, Osteoporosis | Indapamidum, Pantoprazolum ,Sotalolum, Trimetazidini dihydrochloridum, Acidum acetylsalicylicum, Simvastatinum, Amlodipinum, Naproxenum, Cholecalciferolum, Formoteroli fumaras, Tizanidinum, Theophyllinum, Fluticasoni propionas, Salmeterolum, Tiotropium, Pantoprazolum |
| 10 | 79 | Female | 28.0 | Nuclear/Cortical/Subcapsular | Hypertension, Coronary Artery Disease, Hyperlipidemia | Perindoprilum argininum, Bisoprololi fumaras, Lacidipinum, Simvastatinum, Piracetamum, Acidum acetylsalicylicum |
| 11 | 78 | Male | 29.1 | Nuclear/Cortical | Hypertension | Bisoprololi fumaras |
| 12 | 78 | Male | 21.3 | Nuclear/Cortical | History of Acute Pancreatitis | none |
| 13 | 78 | Male | 24.2 | Nuclear/Cortical | Hypertension, Benign Prostatic Hyperplasia, Atherosclerosis of lower Extremities | Propafenoni hydrochloridum, Pentoxifyllinum, Simvastatinum, Bencyclani fumaras, Tamsulosini hydrochloridum, Finasteridum |
| 14 | 78 | Female | 32.4 | Nuclear/Subcapsular | Asthma | Tiotropium |
| 15 | 77 | Male | 29.4 | Nuclear/Cortical | Hypertension, Coronary Artery Disease, Hyperlipidemia, Gout, Ulcerative Colitis, Chronic Obstructive Pulmonary Disease, history of Bypass Graft due to Leriche Syndrome | Valsartanum, Bisoprololi fumaras, Pentoxifyllinum, Amlodipinum, Atorvastatinum, Nicergolinum, Acidum acetylsalicylicum |
| 16 | 75 | Female | 28.9 | Nuclear/Subcapsular | Parkinson’s Disease, Atrial Fibrillation | Drotaverini hydrochloridum, Metoprololi tartras, Levodopu, Benserazidum |
| 17 | 75 | Female | 15.1 | Nuclear | Hypertension, Coronary Artery Disease, Osteoporosis, Atrial Fibrillation, history of Stroke | Clopidogrelum, Spironolactonum, Metoprololi succinas, Warfarinum natricum |
| 18 | 74 | Female | 23.2 | Subcapsular | Hypothyroidism, Insomnia | Levothyroxinum natricum, Zolpidemi tartras |
| 19 | 74 | Female | 26.0 | Cortical/Subcapsular | Hypertension, Hyperlipidemia | Indapamidum, Bisoprololi fumaras, Perindoprilum argininum, Losartanum kalicum, Simvastatinum, Hydroxyzini hydrochloridum |

Standard topical treatment before cataract surgery included: Ofloxacinum/Moxifloxacinum, Proxymetacaini hydrochloridum, Tropicamidum, Phenylephrinum, Timololum, Povidonum iodinatum and Hydroxyzini hydrochloridum orally.

Table S3. Metabolites differentiating the XFS group from the control group.

| Name | CV [%] | Monoisotopic mass [Da] | RT [min] | Fragments [m/z] | Types of chromatography | Ionization |
| --- | --- | --- | --- | --- | --- | --- |
| L-Serine | 10 | 105.0426 | 8.8 | 74.0247, 104.0351 | Hilic (neg) | M-H |
| 3-Hydroxy  anthranilic acid | 22 | 153.0426 | 5.0 | 53.0386, 80.0494, 108.0439, 136.0388 | Hilic (pos) | M+H-H2O |
|  | 13 | 153.0426 | 4.9 | 80.0495, 108.0444, 136.0394 | C18 (pos) | M+H-H2O |
| Indoleacetaldehyde | 2 | 159.0684 | 7.0 | 117.0564, 145.0505, 160.0749 | Hilic (pos) | M+H |
| 2-Hydroxycinnamic acid  /m-Coumaric acid  (co-elution) | 4 | 164.0473 | 2.8 | 77.0365, 91.0522, 93.0674, 95.0474, 103.0522, 121.0620, 123.0416, 147.0412, 165.0519/  65.0351, 91.0522, 119.0469, 147.0412, 165.0519 | C18 (pos) | M+H |
| L-Arginine | 3 | 174.1117 | 11.7 | 60.0560, 70.0652, 116.0703, 130.0969, 158.0919, 175.1188 | Hilic (pos) | M+H |
| Ascorbic acid | 2 | 176.0321 | 6.3 | 85.0284, 95.0127, 113.0230, 129.0168, 141.0160, 159.0894 | Hilic (pos) | M+H |
| Homo-L-arginine | 16 | 188.1273 | 12.0 | 60.0558, 84.0807, 126.1021, 130.0862, 144.1127, 172.1077, 189.1344 | Hilic (pos) | M+H |
| Ergothioneine | 20 | 229.0884 | 1.1 | 60.0805, 127.0316, 186.1051, 230.0950 | C18 (pos) | M+H |
|  | 3 | 229.0885 | 9.2 | 60.0813, 86.0040, 100.0219, 127.0319, 186.1054, 230.1568 | Hilic (pos) | M+H |
| Hydroxybutyrylcarnitine | 16 | 247.142 | 1.2 | 60.0801, 85.0279, 124.0870, 144.1008, 202.1051, 230.0418, 248.1494 | C18 (pos) | M+H |
| Decatrienoylcarnitine | 8 | 309.194 | 5.6 | 60.0802, 85.0284, 144.1009, 251.1263, 310.2003 | C18 (pos) | M+H |
| S-adenosyl-L-methioninate | 8 | 398.1372 | 13.4 | 102.0534, 136.0614, 250.0925, 399.3031 | Hilic (pos) | M+H |
